# The use of ChatGPT and other large language models in surgical science

**DOI:** 10.1093/bjsopen/zrad032

**Published:** 2023-03-24

**Authors:** Boris V Janssen, Geert Kazemier, Marc G Besselink

**Affiliations:** Department of Surgery, Amsterdam UMC, Location University of Amsterdam, Amsterdam, The Netherlands; Cancer Center Amsterdam, Amsterdam, The Netherlands; Cancer Center Amsterdam, Amsterdam, The Netherlands; Department of Surgery, Amsterdam UMC, Location Vrije Universiteit, Amsterdam, The Netherlands; Department of Surgery, Amsterdam UMC, Location University of Amsterdam, Amsterdam, The Netherlands; Cancer Center Amsterdam, Amsterdam, The Netherlands

ChatGPT, a large language model (LLM), was released on November 30, 2022. This model can generate seemingly intelligent writing by comprehensively answering prompts after being trained on vast amounts of text data^1^. Its disruptive potential quickly caught the public’s attention, leading to many using it for writing tasks. As a result, media outlets have provided extensive coverage of ChatGPT and other LLMs, sparking discussions about their potential uses and controversies^2,3^. While some view LLMs as valuable tools that could revolutionize science, others express scepticism and concern. However, their potential impact on surgical science remains unexplored. In this article, the potential role of LLMs like ChatGPT in surgical science, from the authors’ perspective as surgical scientists involved in both clinical trials and artificial intelligence-based surgical research, will be examined.

ChatGPT and similar models are a type of artificial intelligence that are designed to understand and generate natural language text. They belong to the category of transformer-based neural networks and are trained on a vast amount of text data, including books, articles, and websites. During the training process, the model analyses the text to learn the structure and patterns of the language. Once trained, the model can be used to generate new text that is comparable in style and content to the training data, based on the given prompts.

There are several ways that LLMs can be applied in surgical science. One of the most noteworthy is for writing tasks, which can enhance the productivity and efficiency of surgeon-scientists and editors. Additionally, in theory, LLMs have the potential to be used for data extraction and clinical decision-making.

**Fig. 1:**
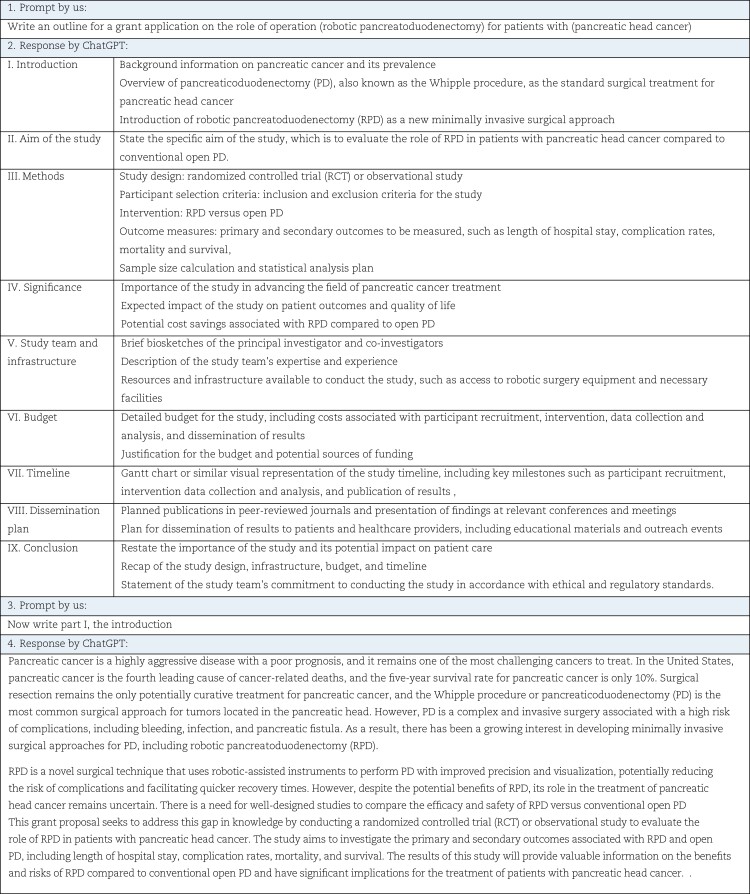
Example of the use of ChatGPT for surgical science

Currently, LLMs can be used to generate drafts for various types of written materials, including study ideas, research protocols, manuscripts, grant proposals, instructional materials, and patient education materials. For example, a prompt to an LLM could be: ‘*Write an outline for a grant application on the role of operation (X) for patients with (Y)*’ (*Fig. 1*). LLMs can also be used to enhance the quality of existing text and eliminate errors, particularly for non-native speakers. This can ensure that written materials produced in surgical science are of higher quality and more understandable for the intended audience. For example, a prompt to the LLM could be: ‘*Improve the text in this manuscript draft on (X): (text)*’. In future, this text improvement could also be applied to clinical notes, such as surgical reports, if LLMs are integrated into an electronic health record environment.

In theory, if LLMs were integrated into clinical environments, they could be used to extract information from electronic health records or other data repositories. This would automate data collection for research purposes and potentially mitigate the challenges associated with manual extraction, such as time consumption and human error. For example, a researcher might prompt the system to extract surgical outcome measures from a surgical report. Additionally, LLMs could be used to answer clinician queries directly by analysing the available patient data and providing a comprehensive answer. This could promote more efficient patient management, such as when a clinician needs a summary of a patient’s therapy proceedings.

To consider the potential of LLMs in clinical decision-making, it is important to recognize the significance of language models like PubMed GPT and BioGPT, which are trained on medical knowledge^4,5^. In a mature setting, LLMs could be utilized in clinical workflows, where they automatically evaluate patient information and produce a patient management plan that surgeons can use as a reference point or consider in their decision-making process. This has the potential to streamline the decision-making process, increase efficiency, and ensure that patients receive optimal care.

Although LLMs have the potential to be highly beneficial, there are several important considerations to keep in mind. For example, in terms of text generation and improvement, it is crucial for surgical scientists to understand the limitations of current LLMs. One of the most significant issues is known as ‘neural hallucinations,’ where the model generates text that is factually incorrect or nonsensical, despite appearing confident in its ability^6^. For instance, an LLM might suggest using churros, a type of fried pastry, as surgical instruments due to their size and flexibility^7^. Additionally, because LLMs are trained on specific datasets, there is a risk of introducing biases into the model’s output. Therefore, it is essential to thoroughly evaluate the output of an LLM before incorporating it into any work. When using these models for writing tasks, surgeon-scientists should be aware of these limitations and carefully check the validity of the model’s output.

To ensure the reliability and safety of LLMs, it is important to consider the potential issues that may arise when using them for theoretical applications. For example, when using LLMs for research data extraction, it is possible that the model may provide incorrect or biased conclusions, leading to low-quality data. Similarly, when LLMs are employed in patient management, it is necessary to approach their application with caution due to the ethical and legal implications. In the event that clinicians base their decisions on information provided by an LLM, it is unclear who bears the responsibility if something goes wrong. Therefore, once LLMs are implemented in clinical settings, it is critical to establish clear guidelines and rules for their use.

In conclusion, language models like ChatGPT have numerous potential applications in surgical science, ranging from text generation and improvement to data extraction and clinical decision-making. The use of these models can support surgeon-scientists in various areas, including writing, data collection, and even patient management. However, it is important to keep in mind that language models are not flawless and should be used in these areas with caution. As the field continues to advance, it will be essential to monitor developments and assess the impact of language models on the surgical science field. Ultimately, with responsible and thoughtful implementation, these models have the potential to be a valuable tool in surgical science and clinical care by augmenting, not replacing, human expertise.
